# Mechanical Characterisation of Bond Formation during Overprinting of PEEK Laminates

**DOI:** 10.3390/ma17010161

**Published:** 2023-12-28

**Authors:** Simon Hümbert, Fynn Atzler, Heinz Voggenreiter

**Affiliations:** German Aerospace Center (DLR), Institute for Structures and Design (BT), 70569 Stuttgart, Germany

**Keywords:** additive manufacturing, FDM, overprinting, PEEK, thermoplastic composites, in situ bonding, automated fibre placement

## Abstract

The latest generation of high-temperature 3D printers enables the production of complex structural components from aerospace-grade thermoplastics such as PEEK (polyether ether ketone). However, adding long or continuous fibres is currently limited, and thermal stresses introduced during the process restrict the maximum part dimensions. Combining 3D-printed components with continuous fibre-reinforced components into one hybrid structure has the potential to overcome such limitations. This work aims to determine whether in situ bonding between PEEK laminates and PEEK 3D printing during overprinting is feasible and which process parameters are significantly responsible for the bonding quality. To this end, the bonding is analysed experimentally in two steps. Firstly, the influence of the process parameters on the thermal history and the strength of the bond is investigated. In the second step, a detailed investigation of the most critical parameters is carried out. The investigation showed the feasibility of overprinting with bonding strengths of up to 15 MPa. It was shown that the bonding strength depends primarily on the temperature in the interface. Additionally, the critical parameters to control the process were identified. The process influences that were displayed form the basis for future hybrid component and process designs.

## 1. Introduction

In recent years, there has been notable progress in the additive manufacturing of high-temperature thermoplastics, such as PEEK (polyether ether ketone) or PEI (polyethyleneimine), through material extrusion processes like Fused Filament Fabrication (FFF) or Fused Granular Fabrication (FGF). Various solutions for thermal management and an exemplary implementation were shown by [1]. This printing process has become a standard procedure for producing complex parts. Studies have shown that high process temperatures are necessary for adequate interlayer bonding and, subsequently, proper mechanical properties of high-temperature printed parts [2,3]. To cater to this need, the newest generations of commercial, high-temperature FFF machines have heated print beds and chambers that can reach up to 300 °C, making it possible to manufacture high-temperature, aerospace-grade materials through additive manufacturing.

Although 3D-printed, high-temperature thermoplastic material has promising properties, its mechanical performance still falls short of that achieved through injection moulding [4]. Reviews by [4] and [5] have shown that the reduction in mechanical properties of 3D-printed material compared to other manufacturing processes is mainly due to low inter-layer bonding strength and porosity. Additionally, generated thermal stresses during material extrusion can result in warpage and fractures within the part, thereby limiting the part’s maximum dimensions [5,6]. These factors, together with the limited ability to add long or even continuous fibres [5], still severely restrict the use of additive manufacturing of thermoplastics for aerospace applications.

To overcome these limitations in manufacturing, one possible solution is to merge thermoplastic 3D printing with automated fibre placement or compression moulding. This combination aims to combine the intricate design possibilities of 3D printing with the exceptional material properties of continuous fibre-reinforced thermoplastics and their high production output.

One approach to achieving this goal is to produce continuous fibre-reinforced laminates and 3D-printed components separately and then combine them. Li et al. [7] studied sandwich structures with complex 3D-printed core shapes joined with continuous fibre-reinforced laminates using adhesive bonding. Another option is tailored composite designs like those proposed by Janssen et al. [8], where consolidation between printed core and laminate occurs through a thermoforming process. Rakhshbahar et al. [9] proposed achieving the bonding during automated fibre placement (AFP). When using AFP to join components, the heat source of the AFP system (e.g., laser) is used to melt the surface of the substrate and thermoplastic tapes. The components are then consolidated using a compaction roller [10].

Other studies have explored the possibility of bonding 3D-printed structures directly onto laminates without additional joining steps, similar to overmoulding. In one example, Morales et al. [11] developed a process called “over 3D-printing” using PA 6 (polyamide 6) organo-sheets. A more detailed analysis of the bond strength between PA 6 laminates and 3D-printed PA 6, especially the influence of process parameters, has been shown by Penter et al. [12] and Maier et al. [13]. Boros et al. [14] conducted a study on PLA (polylactic acid), comparing the properties of overprinting with overmoulding. Raspall et al. [15] describe a facility where two cooperating robots perform the tape placement and printing simultaneously. An automated process chain for the flexible production of hybrid components using non-planar printing using industrial robots has been described by Matkovic et al. [16]. This process chain offers several features to optimise the bonding conditions, such as pre-heating, substrate heating and additional consolidation pressure.

All these studies were conducted with commodity or engineering plastics. Expanding the overprinting process to high-temperature thermoplastics will increase the demands on the process. The main requirements can be derived from the theory of layer adhesion in 3D printing. Sun et al. [17,18,19,20] described the bond formation as a diffusion bonding based on the crack healing theory by [21]. Coogan et al. [22,23,24] and Li et al. [25] have shown a similar approach. An adaption of such a model using PEEK was shown by Basgul et al. [26]. The main principle of these bonding mechanisms is that bond formation occurs only as long as the interface temperature is above the thermoplastic’s glass transition temperature, Tg. Regarding semi-crystalline thermoplastics, the bond formations widely stopped once the interface temperature had fallen short of the re-crystallisation temperature.

The importance of interface temperatures for the 3D printing of PEEK was also shown experimentally. Zanjanijam et al. [27] give an overview of the main challenges of printing PEEK and state that, additionally to the maximum temperatures, minimising the difference between nozzle and substrate temperature is the most critical aspect for sufficient mechanical properties. Similarly, Yang et al. [2] as well as Wang et al. [28] report increasing mechanical properties and an increasing degree of crystallinity for increasing process temperatures. Yi et al. [29] investigated the correlation between crystallinity and mechanical properties and showed the importance of controlling the crystallinity. Pu et al. [30] have shown similar effects for dynamic mechanical analysis. Deng et al. [31] and Wu et al. [32] described the significant effects of layer thickness and infill. These results not only represent the main requirements for FFF printing of PEEK but can also be largely applied to the overprinting of PEEK laminates.

First experimental studies on bonding effects between printed PEEK and PEEK laminates during overprinting have been presented by Caprais et al. [33,34] and Hümbert et al. [35]. The results support the claim that high bonding temperatures are crucial for bonding strength.

The German Aerospace Center’s Institute for Structures and Design (BT) is working on integrating 3D printing into the process chain for producing thermoplastic composites. The aim is to use 3D printing to increase the complexity and functionality of the structures. Overprinting of laminates made of high-performance thermoplastics plays a central role here. Ref. [36] shows an example of combining 3D printing and automated fibre placement in two robotic cells using PEEK. Other applications are the integration of electronics into sandwich structures [37,38] or the application of radiation protection for space applications [39,40].

While the basics of making hybrid components by overprinting thermoplastic laminates have been described, a deeper understanding of the process is necessary to use the method for high-performance applications. Thus, the major goal of this work is the characterisation of bonding mechanisms between the 3D-printed structure and the laminate during overprinting. Short-fibre-reinforced PEEK is considered for overprinting CF-PEEK laminates to enable the application of this process to primary aerospace structures. To achieve this goal, the investigation is divided into two steps. First, a parameter screening is carried out to identify the crucial elements that influence the bonding between the 3D print and the laminate. The bond’s strength and the interface’s thermal history are analysed. In the second step, a detailed characterisation of these crucial influencing factors is performed.

## 2. Materials and Methods

**Figure 1:**
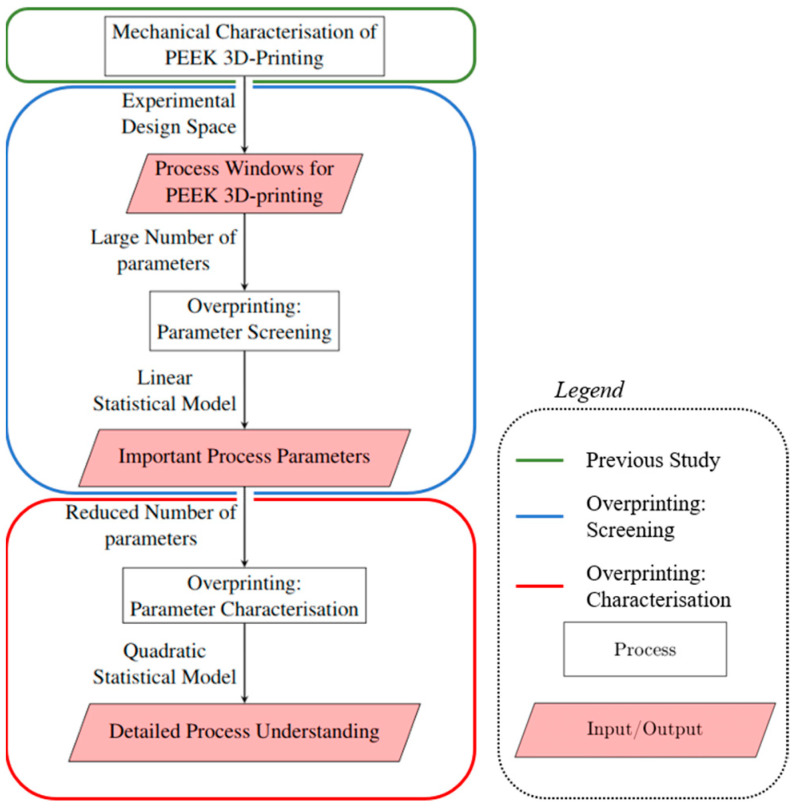
Flow chart of the structure of the investigation [33].

Additive manufacturing is characterised by a large number of process parameters that have a complex effect on the properties of the finished part. During overprinting thermoplastic laminates, both the requirements for the joint between the laminate and the 3D-printed component and the requirements for the 3D-printed component itself must be met. Therefore, overprinting should be carried out within the process window for PEEK 3D printing. This process window was determined experimentally in a previous study [35] and serves as a boundary condition for the experimental design space in this work. The workflow used within this work and discussed above is visualised in Figure 1.

The parameter screening of the overprinting process is supposed to identify the most significant effects of process parameters on the in situ bonding of printed PEEK onto laminates using minimal testing effort. The results from the preliminary study serve as the limits for the test design.

Based on the results of the parameter screening, the test design was narrowed down for a final characterisation of the overprinting process. The characterisation described not only the main effect of the parameters but also the interactions of these parameters, providing a detailed understanding of the overprinting process.

### 2.1. Materials and Printer

**Figure 2:**
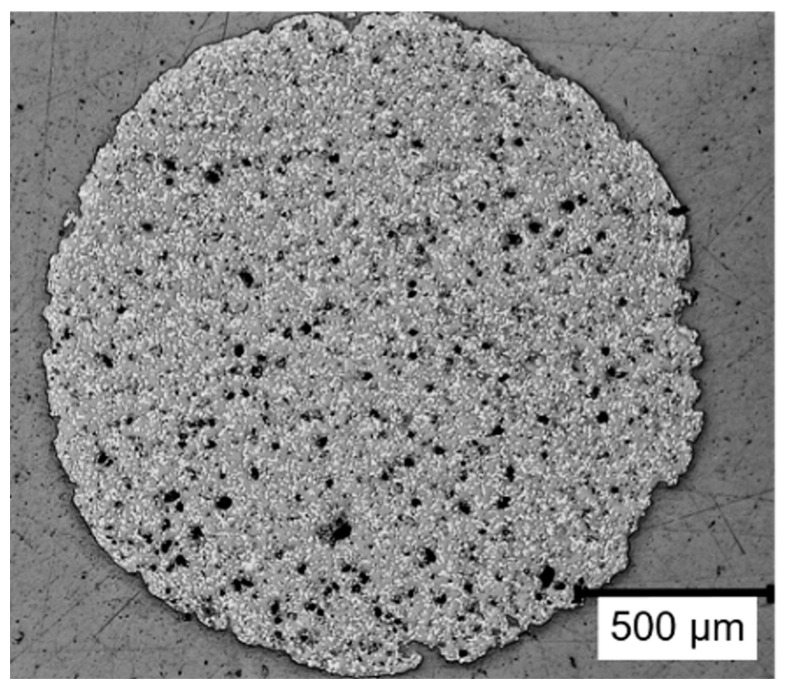
Microsection of PEEK-CF30 filament.

All specimens for the screening and characterisation of overprinting were printed from Ensinger TECAFIL PEEK VX CF30 (Ensinger Plastics, Nufringen, Germany) [41], a PEEK filament filled with 30 wt% carbon fibres. The carbon fibre filling primarily improves processability. On the one hand, a stiffer filament and a higher melt viscosity ensure a stable material extrusion. On the other hand, the fibre content reduces heat shrinkage. At the same time, the fibre content also increases the mechanical properties of the printed component. The material properties, as well as the recommended printing parameters from the data sheet, are summarised in Table 1. A microsection of the used filament is shown in Figure 2. The laminate used for overprinting was a Toray Cetex 1200 UD laminate (Toray Advanced Composites, Morgan Hill, CA, USA) in the configuration [45/0/−45/90]_s_.

For this study, all specimens were printed using a GEWO HTP-260 (GEWO3D, Wörth, Germany) [42] high-temperature FFF printer. The printer is equipped with a heated bed that can reach up to 270 °C, a heated chamber that can reach up to 260 °C, and a nozzle that can be heated up to 450 °C.

### 2.2. Experimental Design

In a previous study [35], the influence of process temperatures on the mechanical properties of printed PEEK was investigated. It was shown that a build chamber temperature of at least 200 °C and a nozzle temperature of at least 430 °C are necessary for acceptable print quality with the given printer. These values serve as lower limits for the experimental design in this work.

The overprinting screening and characterisation test plans were designed using the DoE software Design Expert 13. The software optimises the tested factor levels and fits a surface response model to the experimental results. ANOVA (analysis of variance) was performed to evaluate the fitted model and find the significant effects. Model parameters and effects were considered significant with a *p*-value below 0.05. Results are represented by the model prediction at the centre point of the design and the 95% confidence level. The intensity of effects is described by the Pearson correlation r. Pearson correlation r describes the nature and strength of a linear effect with a single value between −1 and 1.

#### 2.2.1. Overprinting: Parameter Screening

The screening consists of 12 runs fitting a linear surface response model. Response Surface Methodology (RSM) allows a large number of process parameters to be evaluated with relatively few tests. This linear model is designed to find the main effects of the process parameters on the bond strength. Process temperatures were investigated along with print speed, extrusion factor and the height of the first printing layer, as these parameters will likely impact the thermal history in the bonding interface. Additionally, an over-extrusion of the first printing layer was expected to increase the pressure and surface wetting during bond formation. The used parameter ranges and the parameters for each run for printing are shown in Table 2. The entire test design is shown in Table A1 in Appendix A. The bond strength was measured using guided compression shear tests. Additionally, microsections were taken from one specimen per parameter set. The specimen preparation is described in Section 2.3.

#### 2.2.2. Overprinting: Parameter Characterisation

The overprinting characterisation consists of 15 test runs fitting a quadratic surface response model. This model allows the analysis of main effects and interaction effects. A quadratic surface response model is also suitable for optimising the process. However, the optimisation is not the subject of this work. Since the separation of substrate temperature and chamber temperature has turned out unsuitable with the given printer setup, both temperatures are summarised into ambient temperature, with both temperatures at the same value. The investigated parameters were the nozzle temperature, ambient temperature and the height of the first printing layer. The print parameters are shown in Table 2. The entire test plan is shown in Table A2 in Appendix A. Specimen preparation microsections were carried out as described in Section 2.3. Additionally, the first layer height was measured using a 3D optical profilometer.

### 2.3. Test Procedure and Equipment

#### 2.3.1. Compression Shear Testing

**Figure 3:**
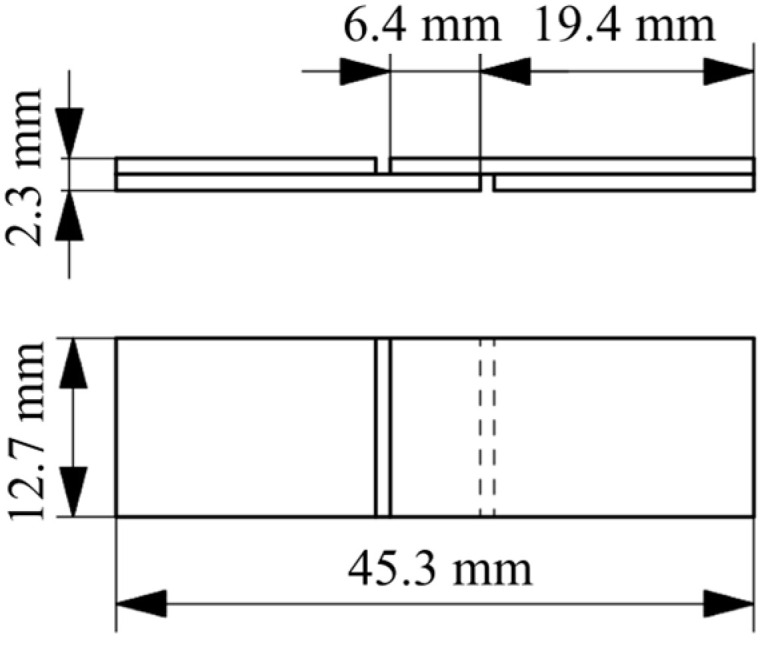
Geometry of compression shear specimens based on ASTM D3846-08 [43].

To qualify the bond strength between 3D printing and laminates, the inter-layer shear strength was measured using single lap shear specimens based on ASTM D3846-08 [43]. In contrast to other frequently used ISO standards, this ASTM standard provides a fixture to guide the specimens. The guided compression test was used to avoid buckling and peel loads in the interface. Due to the asymmetric stiffness of the specimens created by the short fibre-reinforced 3D print and the continuous fibre-reinforced laminate, non-guided specimens like single-lap tensile tests have proven unsuitable to characterise the bond strength. To further reduce the effect of residual stresses and warping in the specimens, the dimensions were scaled down to the values shown in Figure 3.

The testing machine RetroLine 1494 from ZwickRoell GmbH & Co. KG (Ulm, Germany) was used to determine the mechanical characteristics. Tensile tests were performed using mechanical clamps, and the compression shear tests used a fixture, according to [43].

**Figure 4:**
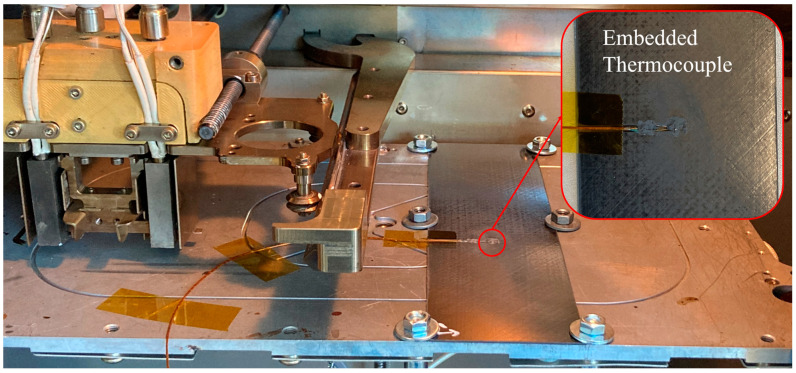
Print setup with embedded thermocouple.

Using this geometry, six specimens per configuration were printed onto one laminate, from which one was used for temperature measurement and the remaining five were used for mechanical testing. The interface temperature was measured using a single thermocouple embedded in the laminate. For this purpose, the laminate was notched halfway and the thermocouple was soldered in so that it was flush with the surface of the laminate. The print setup is shown in Figure 4.

**Figure 5:**
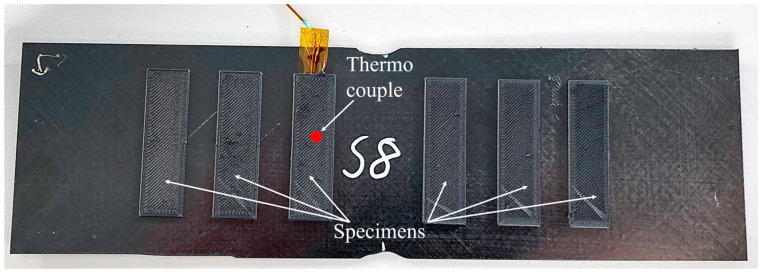
Printed specimens on laminate.

Using this setup, the laminate, including the embedder thermocouple, was overprinted with the six specimens. The specimens were then separated and notched with a disk saw. The overprinted laminate with six specimens is shown in Figure 5.

#### 2.3.2. Microanalysis

The light microscopic examination was performed using the digital VHX-5000 from Keyence Deutschland GmbH (Leinfelden-Echterdingen, Germany) [44]. The objective used was the dual zoom objective VH-ZST with 20 to 200× or 200 to 2000× magnification.

The height profiles were measured using the Keyence vr-5200 optical 3D profilometer. The device achieves a measurement accuracy of ±2.5 µm [45].

## 3. Results

Test results are presented in two steps: parameter screening and characterisation.

### 3.1. Overprinting: Parameter Screening

**Figure 6:**
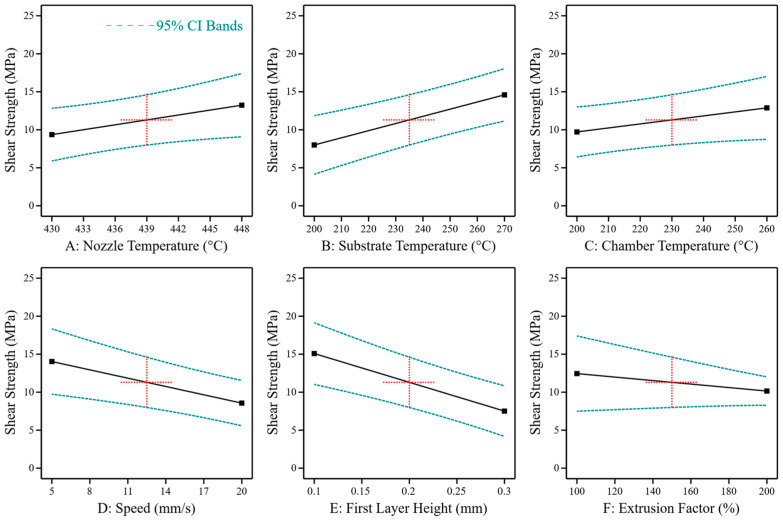
Main effects of process parameters on the shear strength of bonding between PEEK 3D print and PEEK laminate (extended from [35]). The black line shows the predicted effect. The blue lines show the 95 % confidence interval. The model was evaluated at the centre points of the test room (red crosses).

The main effects of the tested process parameters and the 95% confidence band of the used linear effect model are shown in Figure 6. Similar to the material properties of printed PEEK, higher process temperatures increase the bond strength. A significant effect can be observed for the nozzle temperature (*p* = 0.036, r = 0.127) and the substrate temperature (*p* = 0.0001, r = 0.537) generated by the heated print bed. The effect of the chamber temperature is statistically not significant, with *p* = 0.071. Besides the process temperatures, print speed (*p* = 0.002, r = −0.135) and first layer height (*p* < 0.0001, r = −0.460) are significant and thereby, the distance between the hot nozzle and the bonding zone shows significant effects. The effect of the extrusion factor is not significant at *p* = 0.193.

**Figure 7:**
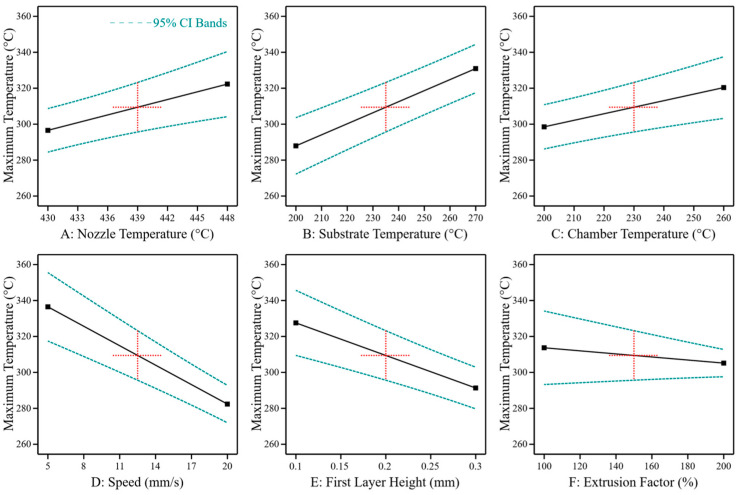
The main effects of process parameters on the maximum bonding temperature between PEEK 3D print and PEEK laminate. The black line shows the predicted effect. The blue lines show the 95 % confidence interval. The model was evaluated at the centre points of the test room (red crosses).

To better understand the impact of process parameters on the thermal conditions in the bonding zone and the correlation between bonding temperature and bonding strength, the temperature on the top surface of the laminate was measured during each print job. The maximum temperatures reached in the bonding zone are shown in Figure 7. Like the effects on shear strength, process temperatures strongly affect the bonding temperature (nozzle temperature: *p* = 0.036, r = 0.200; substrate temperature: *p* < 0.0001, r = 0.064; chamber temperature: *p* = 0.015, r = 0.236). Additionally, reducing the print speed increases the bonding temperature (*p* < 0.0001, r = −0.362), as well as smaller first layer heights (*p* < 0.0001, r = −0.312). A significant effect can also be shown for the extrusion factor (*p* = 0.014, r = 0.361).

**Figure 8:**
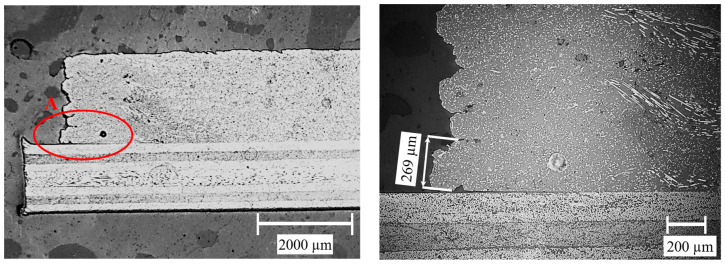
Microsection of a shear specimen with measured first layer height in section A.

Overall, the measurements are subject to large fluctuations within the individual factor combinations. This is shown, among other things, by the wide confidence intervals. The microsections produced provide an indication of a possible cause of the fluctuations. The actual layer height of the first layer does not always correspond to the set target value. This is shown as an example in Figure 8.

**Figure 9:**
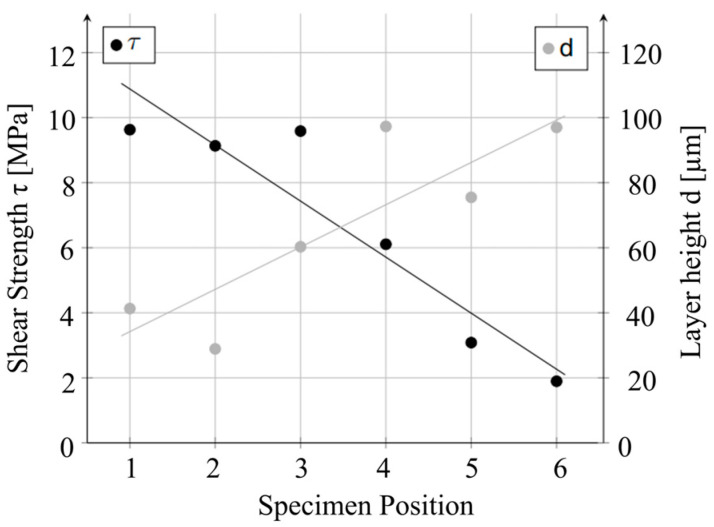
Correlation between bonding strength and tolerance of first layer height depending on the specimen position: 1—left to 6—right (compare Figure 5).

The deviation in the first layer height is due to the fact that the bed levelling, i.e., the setting of the distance between the print bed and the nozzle, is subject to a certain tolerance. On the one hand, this concerns the setting of the zero point of the Z-coordinate. With the printer used, this tolerance is in the range of 0.05 mm and can significantly influence the layer height. Secondly, the parallelism between the print bed and the movement plane of the nozzle plays an important role. Due to a tolerance of this parallelism, the layer height of the first layer can fluctuate across a laminate. An example of the effect of a non-parallel laminate is shown in Figure 9. In this example, the first layer height for each specimen of a configuration was determined using microsections and displayed together with the corresponding strengths of the specimen. A correlation between the position of the sample and the strength is clearly recognisable.

### 3.2. Overprinting: Parameter Characterisation

Based on the results from the screening, three parameters were chosen for closer investigation. The substrate temperature showed the strongest correlation overall, with a Pearson coefficient of r = 0.537. Together with the nozzle temperature, these two factors directly influence the temperature in the bonding zone and, thus, the strength. Therefore, nozzle and bed temperatures were included in the characterisation. The screening tests have shown that individual settings for substrate (print bed) temperature and chamber temperature are very limited with the given 3D printer, as the heating power does not allow large differences between the two parameters. Therefore, the chamber temperature is set to the same value as the substrate temperature and both parameters are combined as one ambient temperature. The second strongest effect was measured for the first layer height with r = −0.460. Consequently, the third factor investigated is the first layer height.

**Figure 10:**
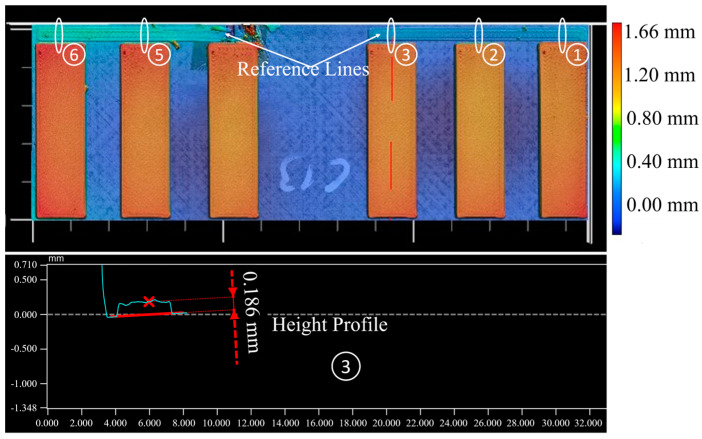
Measurement of first layer height using a 3D optical profilometer.

Furthermore, the screening has shown that the tolerance of the first layer height is difficult to control. It therefore makes sense to measure the first layer height of each specimen individually in order to avoid errors in the evaluation. However, the microsections did not prove to be a reliable measurement method, as the boundary between the first and second layer is not always clearly recognisable. For this reason, the first layer height was measured using a 3D optical profilometer. For this purpose, a reference line with a single layer height was printed along all specimens and analysed at the position of each sample. The measurement concept is shown in Figure 10.

**Figure 11:**
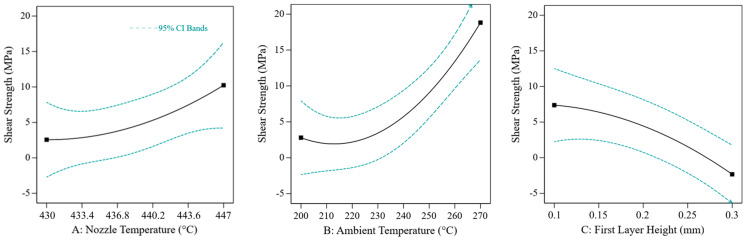
Main effect plots of the characterisation on shear strength. The black line shows the predicted effect. The blue lines show the 95% confidence interval.

The measurements with individually adjusted layer height were then used to create a quadratic model of the effects of the three factors. The model shows significant effects for all three factors (nozzle temperature: *p* = 0.0057; ambient temperature: *p* < 0.0001; first layer height: *p* = 0.0002). The effect plots are shown in Figure 11. In particular, the ambient temperature and the layer height have a non-linear effect. An increase in the ambient temperature, therefore, increases the shear strength disproportionately, while an increase in the layer height reduces the strength disproportionately. Regarding the Pearson correlation, the effects of the ambient temperature (r = 0.567) and the layer height (r = −0.579) are similarly strong. On the other hand, the effect of nozzle temperature is notably weaker (r = 0.325).

**Figure 12:**
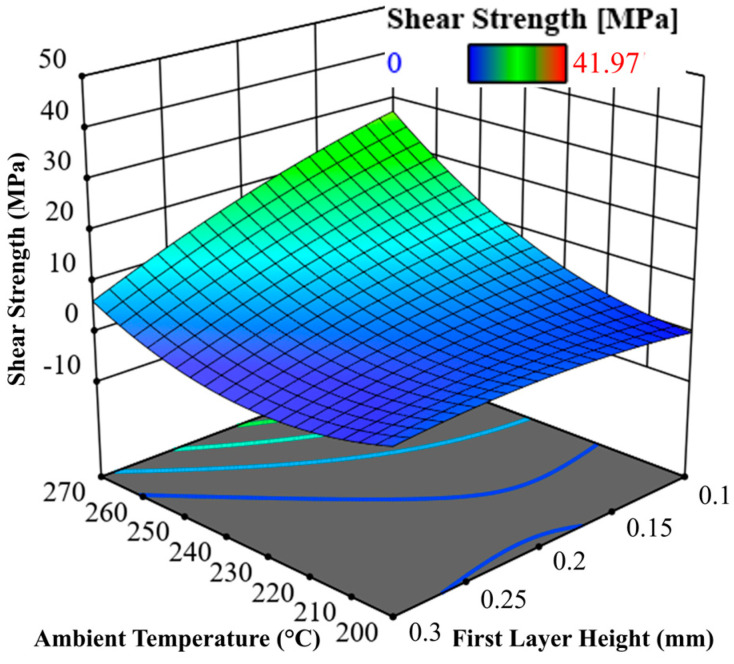
3D plot of the interaction effect between first layer height and ambient temperature.

In addition to the main effects, the quadratic model also allows the effect interactions to be analysed. The interactions between nozzle temperature and ambient temperature (*p* = 0.2934), as well as nozzle temperature and layer height (*p* = 0.1165), are not significant. Only the interaction between ambient temperature and layer height is statistically significant (*p* < 0.0001). This interaction is shown in Figure 12. At high first layer heights, the ambient temperature has no clear effect. However, the effect of the ambient temperature is clearly recognisable at lower first layer thicknesses.

**Figure 13:**
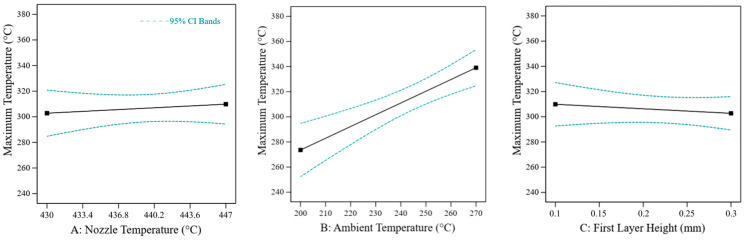
Main effect plots of the characterisation on maximum temperature. The black line shows the predicted effect. The blue lines show the 95 % confidence interval.

In addition to the shear strength, the temperature in the interface between the laminate and 3D print was measured, as in the screening. As the sample size for the temperature measurement was significantly smaller than for the shear tests (only one sample per configuration), only a linear statistical model was used for the maximum temperatures. It was also not possible to analyse the first layer height of the sample for temperature measurement, as the thermocouple interrupts the reference line at this point. Therefore, the first layer height of the neighbouring sample (position 5 in Figure 10) was used for the evaluation. In the resulting model, only the ambient temperature shows a significant effect (*p* = 0.0005; r = 0.879). The effects of the nozzle temperature (*p* = 0.5528) and the first layer height (*p* = 0.4830) are not significant. The effect plot for the maximum interface temperature is shown in Figure 13.

## 4. Discussion

The parameter screening shows significant effects of the process temperatures, print speed, and first layer height on the bond strength between printed PEEK and the laminate. Furthermore, all parameters show an effect on the maximum bonding temperature with very similar magnitudes compared to the effects on bond strength. This indicates that the bonding temperature is the dominant factor for the bond formation between laminates and 3D print. The influence of the process temperatures on the interface temperature thus corresponds to the expectations and observations in the literature on PEEK 3D printing, as these process parameters directly affect the interface temperature. The influence of printing speed and first layer height, however, is indirect. In FFF printers, the print head with the heated nozzle and a heating block forms a strong heat source that affects the process. At a slow printing speed, the heated nozzle remains in the area of influence of the freshly extruded material for longer, which increases the interface temperature. The situation is similar to the layer height. At low layer heights of the first layer, the heated nozzle is closer to the interface, which increases the interface temperature. Increasing the bonding pressure by introducing an over-extrusion showed no additional effect on the bond strength, supporting this theory. The dominant influence of the bonding temperature in the strength is also shown statistically, with a correlation coefficient of *p* = 0.707. These results are in agreement with the bonding theory for 3D printing used by the group of Sun et al. [17,18] and Coogan et al. [22], as well as experimental studies of PEEK 3D printing [27].

The detailed characterisation of the three factors—nozzle temperature, ambient temperature and first layer height—has enabled a better understanding of the effects. The non-linearity of the effects also fits in with the theoretical models mentioned, which are based on crack healing, according to Wool et al. [21]. According to this theory, the temperature of the bonding zone influences the development of strength to the fourth power.

While in the screening all effects on the bonding strength can be attributed to the effect of the process parameters on the interface temperature, this statement cannot be made so easily in the characterisation. In this case, the nozzle temperature and the layer height have a significant effect on the bonding strength but not on the interface temperature. The cause of this observation cannot be conclusively assessed within the scope of this work. While five samples were tested for each parameter combination in the mechanical tests, only one measured value is available for the temperature in the interface. At the same time, the first layer height at the temperature measurement point cannot be determined with sufficient accuracy using the profilometer. The lack of significance could, therefore, be attributed to the small sample size.

The observed interaction effect between ambient temperature and first layer height does not initially match the expected results. The data indicate that this effect is primarily due to the fact that the highest first layer height of 0.3 mm and the lowest ambient temperature of 200 °C are both at the very edge of the process window. The interaction effect is presumably caused by the fact that the main effects in the edge area of the process window can no longer be mapped cleanly.

Overall, the shear tests show high deviations, compromising the quality of the used statistical models. Especially the first layer height, or in other words, the distance of the nozzle from the substrate, has turned out to be a critical factor for the overall process. While the first layer height is a major factor impacting the bond strength, the factor is hard to control. Typical set values of 0.1–0.3 mm of standard printers are within the tolerance range of print bed calibration of the used 3D printer. Additionally, the thickness tolerance of the laminate adds to the tolerance chain of the first layer height. Controlling the tolerance chain of the first layer must be a primary requirement for overprinting laminates, especially when upscaling to large structures.

Another hard-to-control error source is the porosity of the printed material. The filament used to print the samples already shows a porosity of approx. 20% (see Figure 2). In the printed samples, a porosity of a comparable order of magnitude can be observed (see Figure 8). The exact influence of this porosity on the bonding strength cannot be determined within the scope of this work.

## 5. Conclusions

This work shows an analysis of bond formation during the overprinting of continuous fibre-reinforced PEEK laminates with short fibre-reinforced PEEK filament. The analysis is carried out in two steps. Firstly, the influence of the six most important process parameters (nozzle temperature, substrate temperature, chamber temperature, print speed, first layer height, and extrusion factor) on the bonding strength was analysed in an experimental screening. In the second step, the three parameters with the greatest influence were analysed in detail.

The study shows a strong correlation between the process temperatures and the bonding strength. At the same time, it was shown that the toolpath generation, e.g., speed and layer height, also influences the bonding strength. Overall, the temperature and strength measurements showed that the bonding temperature in the interface is the critical factor for successful bonding. This interface temperature is, in turn, influenced by the aforementioned process parameters.

While the concept of overprinting PEEK laminates was proven with an inter-layer shear strength of up to 15 MPa, four major findings can be highlighted as guidance for future process development:

- Bonding temperature in the interface is the primary factor influencing the bond strength.
- Nozzle temperature, substrate temperature, and first layer height are the most important parameters for optimising the bonding strength.
- Substrate pre-heating by means of print bed and chamber heating above 200 °C is required to enable in-situ bonding.
- Controlling the tolerance chain of the first layer height is a crucial requirement to ensure a stable process.

Overall, the results shown can form the basis for future process development and upscaling of the overprinting process using PEEK.

## Figures and Tables

**Table 1:** Material properties of pure PEEK and carbon fibre-reinforced PEEK filament [41].

Property	PEEK-CF30
Density [g/cm^3^]	1.38
Young’s modulus [MPa]	17,500
Ultimate strength [MPa]	190
Glass transition temperature [°C]	143
Melting temperature [°C]	343
Service temperature [°C]	260

**Table 2:** Process parameters of the parameter screening and characterisation.

Process Parameter	Screening	Characterisation
Nozzle Temperature [°C]	430–450	430–450
Substrate Temperature [°C]	200–270	200–260
Chamber Temperature [°C]	200–260	200–260
Speed [mm/s]	5–20	12.5
First Layer Height [mm]	0.1–0.3	0.1–0.3
Extrusion Factor [%]	100–200	100
Nozzle Diameter [mm]	0.4	0.4
Layer Height Other Layers [mm]	0.2	0.2
Wall line count	2	2
Infill Raster [°]	±45	±45

## Data Availability

The data presented in this study are available on request from the corresponding author.

## Appendix A

**Table A1:** Experimental design parameter screening.

Run	Nozzle Temp. [°C]	Substrate Temp. [°C]	Chamber Temp. [°C]	Speed [mm/s]	Layer Height [mm]	Extrusion Factor [%]
1	430.0	200	200.0	8.6	0.3	100
2	430.0	235	260.0	12.5	0.3	150
3	447.0	230	252.5	5.0	0.3	200
4	447.0	240	200.0	5.0	0.3	200
5	430.0	200	200.0	5.0	0.1	200
6	447.0	235	260.0	5.0	0.1	100
7	447.0	270	252.5	20.0	0.3	100
8	447.0	235	260.0	20.0	0.1	200
9	434.5	230	252.5	20.0	0.1	100
10	430	244	200	20	0.1	100
11	448	200	200	20	0.3	100
12	439	250	207.5	5	0.2	100

**Table A2:** Experimental design parameter characterisation.

Run	Nozzle Temperature [°C]	Ambient Temperature [°C]	Layer Height [mm]
1	439	150	0.2
2	432	260	0.1
3	447	260	0.2
4	432	260	0.3
5	430	150	0.3
6	447	161	0.1
7	447	162	0.3
8	430	206	0.2
9	442	226	0.1
10	430	150	0.1
